# International perspectives on AI-driven pharmaceutical IP challenges

**DOI:** 10.3389/fdgth.2026.1774406

**Published:** 2026-06-22

**Authors:** Grace Y. Wang, Jian-Ming Hao, Joe G. Chen

**Affiliations:** 1 West Windsor High School-North, West Windsor, NJ, United States; 2 Intellectual Property Department, Fox Rothschild LLP, Princeton, NJ, United States

**Keywords:** artificial intelligence, copyright, inventorship, international perspective, drug discovery, intellectual property, patent law, personalized medicine

## Abstract

Artificial Intelligence (AI) is transforming the pharmaceutical sector by optimizing care delivery and accelerating drug design. While AI introduces significant benefits, it presents complex challenges regarding intellectual property (IP) frameworks. This review examines AI-driven innovations in personalized medicine and drug design, with a specific focus on inventorship issues that directly affect pharmaceutical patent protection. We analyze recent United States (US) legal developments concerning AI inventorship, including the US Supreme Court's March 2026 denial of certiorari in Thaler v. Perlmutter, which definitively resolved the question of AI inventorship under US law, provide comparative analyses of approaches in Germany and China, and identify specific lessons, rather than wholesale reforms, that may help stakeholders navigate the evolving intersection of AI and pharmaceutical IP.

## 1 Introduction

Artificial Intelligence (AI) is the simulation of human intelligence within machines and computing systems, modeling complex cognitive processes such as learning, problem-solving, and language processing. The origin of AI traces back to 1950, when Alan Turing published “Computing Machinery and Intelligence” (1), considering how machines could imitate human behavior and thinking, which would later serve as the foundation for the emerging field. The discipline evolved rapidly with increased computational power from the 1990s to early 2000s. Today, AI is dramatically transforming the way humanity experiences the world, and its impact can be felt across societal sectors, including legal, education, healthcare systems, and others. In particular, AI has become increasingly integrated within the biomedical industry, reshaping the way medical organizations operate, especially with machine learning algorithms that accelerate and improve the success rate of the drug discovery process (2, 3).

As healthcare AI innovations continue to make their way through the development process and become increasingly adopted within the biomedical sector, intersections with the legal sector, specifically intellectual property (IP), come into play. Like many other creators, AI innovators desire to reap the benefits of IP, seeking IP protection to secure exclusivity and competitive advantage. In particular, the question of inventorship – who qualifies as the legal inventor of an AI-assisted pharmaceutical discovery – has emerged as a central challenge. The challenge, in the United States (US), comes with fitting the relatively new age of AI into the older, stricter, and more traditional IP framework, which was originally designed to solely accommodate the bounds of human intelligence. Closely related to the inventorship question is the issue of copyright in AI training data: because pharmaceutical AI models depend on large datasets of scientific literature and chemical databases to generate novel drug candidates, the legal status of such training inputs under copyright law directly affects the viability of the AI-driven discovery pipeline whose outputs are subject to patent protection. This review focuses specifically on inventorship issues affecting AI-driven drug discovery and medical applications, with a secondary examination of copyright considerations that bear on the same innovation pipeline. We analyze these issues in depth and detail approaches in the US, Germany, and China, identifying practical lessons that may inform how US stakeholders, including pharmaceutical companies, patent practitioners, and policymakers, approach AI-assisted inventions within existing frameworks.

## 2 Overview of biomedical applications of AI

### 2.1 Personalized medicine

AI can help streamline patient care significantly. Examples include conversational triage and algorithmic dosing. For instance, Buoy Health's chatbot interacts with users in a way that mirrors a doctor-patient dynamic, mimicking real conversations and asking follow-up questions (https://www.buoyhealth.com). As the users answer, the chatbot analyzes the symptoms they are describing and eliminates any conditions that do not match, ultimately sending the user up to three potential diagnoses, along with triage options (4). In this way, patients can receive accurate diagnoses and get a sense of their condition, which may even save them from taking costly, or even unnecessary, trips to the hospital. In an early study, Buoy Health's chatbot proved extremely successful, with researchers finding a decrease in patient uncertainty from 34% to 21% after communicating with the chatbot (4).

Another example is CURATE.AI, an AI platform that identifies optimal treatment doses for individual patients, to target cancer cells and tumors (5). CURATE.AI analyzes minimal patient data, including their responses to previous drug doses, to create a personalized profile and recommend specific chemotherapy doses that match the patient's needs and safety concerns (5). A pilot clinical trial has found that 97% of doses suggested by CURATE.AI have been accepted by clinicians (6). The trial, which involved patients with advanced tumors, demonstrated that the advised optimal doses for some of the patients were roughly 20% lower than the average, suggesting the effectiveness of the algorithm in personalizing treatment dosages (6).

### 2.2 AI-based design of novel medicines

On the other hand, AI-based drug and protein generation holds tremendous potential for the future of biomedical companies. For instance, Chroma, a generative AI model developed by Generate:Biomedicines, has made groundbreaking advancements in designing novel protein sequences and structures that can be tailored towards specific favorable properties and functions (https://generatebiomedicines. com/chroma). Chroma features a diffusion framework respecting statistical distributions of polymers, a neural network for molecular systems that optimizes inter-residue geometries, and layers to efficiently output 3-D protein designs (7). Chroma has experimentally characterized 310 unique proteins that exhibit folded structure and desired biophysical properties, demonstrating the model's tremendous potential in advancing the study of protein generation based on certain criteria (7).

Another cutting-edge discovery AI model is SyntheMol (https://github.com/swansonk14/SyntheMol), developed by a team of researchers at Stanford University. SyntheMol designs novel compounds from chemical space, comprising nearly 30 billion different molecules, to inhibit the growth of Acinetobacter baumannii, a leading Gram-negative bacterial pathogen responsible for deaths related to antibacterial resistance (8). The model integrates building-block libraries, activity data, and reaction templates, and filters out synthetically infeasible compounds. The model essentially hands over chemical recipes to the scientists, so that they can confidently produce structurally new drugs right then and there in the lab. To date, SyntheMol has synthesized 58 novel molecules, 6 of which showed antibacterial activity across pathogens including A. baumannii (8).

Since 2015, approximately 75 AI-discovered drugs have entered clinical trials, with reported Phase 1 success rates of 80−90%, compared to historical rates of ∼40%-65% (9). These promising trends in early trials for AI-discovered drugs suggest that if these rates continue onto Phase 3 and beyond, the probability of a drug completing all phases of clinical trials could increase from 5%-10% to 9%-18%, nearly doubling overall R&D productivity.

From these examples, AI-discovered drugs and other AI-driven inventions will continue to thrive in modern society as time goes on. Given these developments, this review now turns to the core issue: how do current IP frameworks address inventorship for AI-assisted pharmaceutical innovations, and what practical lessons can stakeholders draw from international approaches?

## 3 Current IP issues and policy in the US

### 3.1 Inventorship for AI-assisted inventions

In patents, one of the main categories of IP, defining inventorship is essential. However, AI assistance complicates attribution under human-inventor regimes. The mechanistic nature of healthcare AI, including AI-discovered drugs, clashes with the concept of human inventorship, which patent law is traditionally built upon. An “inventor,” as originally defined by the US Patent Act, is the individual(s) who invented or discovered the subject matter of the invention (10). Courts have interpreted “individual” to mean a natural person. Despite this, some parties still contend that the Constitution does not explicitly state that inventors must be humans.

In 2019, Stephen Thaler filed two patent applications, listing an AI system called “Device for the Autonomous Bootstrapping of Unified Sentience” (DABUS) as the inventor (11). These applications, however, were rejected and labeled as incomplete by the US Patent and Trademark Office (USPTO), with the reasoning being that there was no human inventor accredited. In response, Thaler filed a complaint in a federal district court in Virginia. In 2021, the court ruled in support of the USPTO's decision, citing a previous Supreme Court case in which the label of “individual(s)” referred solely to human beings (11). The court held that the “individual(s)” referred to in the US Patent Act are, likewise, a title limited to natural persons only (11). Thaler later appealed to the Court of Appeals for the Federal Circuit (CAFC), but the CAFC only further affirmed the district court's decisions, thus clarifying that in the US, the label of “inventor” cannot be designated to AI algorithms (12). On March 2, 2026, the US Supreme Court denied certiorari in Thaler v. Perlmutter (Case No. 25-449), declining to review the lower courts’ holdings and thereby bringing definitive finality to the question of AI inventorship under US patent law (13). This decision aligns with the UK Supreme Court's contemporaneous ruling reaching the same conclusion under the UK Patents Act, together establishing a consolidated judicial consensus across major common-law jurisdictions that AI systems cannot serve as legal inventors (14).

These consolidated rulings at the highest judicial levels carry far-reaching consequences for biomedical businesses that have invested heavily in AI, particularly the pharmaceutical companies relying on AI-discovered molecules to accelerate clinical trials and bring new drugs to market. Although the USPTO permits patents involving AI, it does so only when a human has made a significant contribution to the invention (15). While the foundational question of whether AI can be named as an inventor is now definitively settled, practical implementation challenges persist. Empirical data indicates that approximately 75 AI-discovered drugs have entered clinical trials since 2015, with AI-driven processes frequently operating with minimal direct human intervention during key discovery phases (9). This operational reality means that pharmaceutical innovators must carefully document human contributions throughout the AI-assisted discovery process to satisfy the USPTO's significant-contribution standard (15). We note that these judicial decisions reflect a consistent and reasoned interpretation of statutory language. With the legal uncertainty surrounding AI inventorship now resolved by the Supreme Court's denial of certiorari (13), the remaining task for pharmaceutical companies is not to challenge the framework, but rather to develop robust internal protocols for documenting the nature and extent of human contributions at each stage of AI-driven drug discovery workflows, consistent with the USPTO 2024 guidance on AI-assisted inventions (15).

### 3.2 Copyright considerations in AI training

While this review focuses primarily on inventorship, copyright issues in AI training are not a separate concern but rather an upstream element of the same innovation pipeline. Pharmaceutical AI models such as SyntheMol depend on extensive training datasets, including scientific literature, chemical databases, and other materials that may be subject to copyright, to generate the novel drug candidates that are subsequently the subject of patent applications (8). If training on such datasets were found to constitute copyright infringement, the entire AI-driven drug discovery workflow would be jeopardized, regardless of whether the inventorship requirements for the resulting patents are satisfied. Thus, copyright compliance in AI training is a necessary precondition for the lawful operation of the AI systems whose outputs pharmaceutical companies seek to patent. Many AI models are trained on extensive datasets that include scientific literature, chemical databases, and other materials that may be subject to copyright. By learning and analyzing information from these sources, AI algorithms are able to carry out advanced operations, such as discovering novel compounds. When AI training practices come under legal scrutiny, developers often invoke the fair use doctrine as a defense.

Fair use legally permits the usage of copyrighted content without the owner's consent (18). Fair use is evaluated on a case-by-case basis upon four primary factors: “the purpose and character of the use, including whether such use is of a commercial nature or is for nonprofit educational purposes”; “the nature of the copyrighted work”; “the amount and substantiality of the portion used in relation to the copyrighted work as a whole”; and “the effect of the use upon the potential market for or value of the copyrighted work” (16). The fourth market effect factor in particular holds that the new work must not harm the market value of the original material (16). Though all factors are considered when determining fair use applicability, the market effect factor “may be the most important” among the four when determining fair use applicability in technology-related cases (17). For pharmaceutical AI companies, the key takeaway is that training AI models on properly licensed scientific and chemical data, rather than unauthorized materials, remains the most reliable path to avoiding copyright disputes.

## 4 Existing IP policy outside of US

### 4.1 Germany IP policy

As a member of the European Union (EU), Germany operates under EU law. Following his unsuccessful attempts in the US, Stephen Thaler, the creator of DABUS, also sought patents in other jurisdictions, including Germany, naming DABUS as the inventor. In 2024, the case reached the Bundesgerichtshof (BGH), Germany's Federal Court of Justice. Consistent with the position of the European Patent Office (EPO), the BGH held that AI cannot, under any circumstance, be designated as an inventor (14), mirroring the US court's decision. The court reaffirmed that at least some level of human contribution is required, with the natural person credited as the inventor and the AI system treated merely as a tool supporting the inventive process. This framework acknowledges the role of AI in innovation while preserving the traditional principle of human inventorship in law. Notably, however, the German approach diverges from that of the US in one respect: the BGH does not require substantial human involvement. Instead, it is sufficient just to demonstrate that a natural person participated in the inventive activity, even minimally. This lower threshold offers a practical lesson for pharmaceutical companies: in jurisdictions following the German model, documenting any level of human participation in the AI-assisted discovery process may suffice for patent eligibility in these countries. For US practitioners, this distinction highlights the importance of carefully documenting the nature and extent of human contributions throughout AI-driven drug discovery workflows to meet the USPTO's more demanding significant-contribution standard.

Germany has also addressed copyright issues related to AI training data. Under the German Copyright Act, as reinforced by the EU Directive on Copyright in the Digital Single Market, text and data mining (TDM) is permitted for scientific research and, in certain cases, for commercial purposes. This clear statutory exception provides pharmaceutical AI developers operating in Germany with greater certainty when training models on scientific literature and chemical databases. For US pharmaceutical companies, Germany's TDM framework illustrates how explicit statutory exceptions, rather than case-by-case fair use determinations, can reduce legal uncertainty in AI-driven drug discovery.

### 4.2 China IP policy

Over the past few decades, China has emerged as a global hub for AI innovation, with the government placing a strong emphasis on investment in the field. Consequently, the number of AI-related patents, particularly those focused on healthcare applications and AI-enabled drug discovery, has grown exponentially. Although the China National Intellectual Property Administration (CNIPA) continues to stress the necessity of human inventorship, primary concern lies in assessing the tangible impact of the creation, particularly its capacity to solve a technical problem and achieve a concrete technical effect. For example, a simple algorithm is deemed unpatentable (19). However, if an AI model in drug discovery can generate novel compounds and substantially improve the drug screening process, the invention may qualify for patent protection. This technical-effect focus offers pharmaceutical companies an important practical lesson: when seeking patent protection in China for AI-discovered drugs, emphasizing the concrete technical improvements, such as enhanced screening efficiency, novel molecular structures, or improved therapeutic outcomes, may strengthen patent applications. As of 2025, China has become the global leader in AI-related patents, with over 38,000 generative AI patent applications filed between 2014 and 2023, six times the number in the US (20). A significant share of these patents relate to AI-powered drug screening, underscoring the synergy between technical innovation and the patent framework in China.

China has also addressed copyright considerations for AI training. The Interim Measures for the Management of Generative Artificial Intelligence (21), which came into effect in 2023, stipulates that providers of generative AI services must not infringe upon the IP rights, including copyrights, legally held by others. For pharmaceutical AI developers, this reinforces the importance of ensuring proper licensing for training data, consistent with best practices in other jurisdictions.

## 5 Conclusion

AI-driven innovations have transformed industries worldwide, with AI drug discovery emerging as a major force in healthcare. As biotech and pharmaceutical companies embrace these tools to accelerate drug development, they face pressing questions about how to secure patent protection for AI-assisted inventions within existing IP frameworks. The central challenge, inventorship, affects pharmaceutical companies globally, as legal systems maintain the principle that patents require human inventors while AI plays an increasingly autonomous role in the discovery process.

In the US specifically, the Thaler decisions, now conclusively affirmed by the Supreme Court's denial of certiorari in March 2026 (13), have established with finality that AI systems cannot be named as inventors. The UK Supreme Court's parallel ruling under the UK Patents Act reinforces this position across major common-law jurisdictions (14). With this foundational question settled, the USPTO's guidance requiring demonstration of significant human contribution for AI-assisted inventions (15) provides the operative framework for pharmaceutical companies. Examining approaches in Germany and China offers useful lessons: Germany's lower threshold for human participation suggests that documenting any level of human involvement may suffice in some jurisdictions, while China's emphasis on technical effects highlights the value of framing AI-discovered drugs in terms of concrete technical improvements. These comparative insights do not suggest that US law requires fundamental reform, but rather illuminate strategies that pharmaceutical companies can employ to navigate the current landscape.

Overall, the intersection between AI, IP law, and biomedicine is rapidly evolving. For pharmaceutical companies investing in AI-driven drug discovery, the practical imperative is clear: carefully document human contributions throughout the discovery process, secure proper licenses for training data, and tailor patent strategies to the requirements of each jurisdiction. By understanding the different approaches taken by the US, Germany, and China, stakeholders can make informed decisions that protect their innovations while working within established legal frameworks. As AI capabilities continue to advance, ongoing dialogue among innovators, practitioners, and policymakers will help ensure that IP systems remain capable of promoting pharmaceutical innovation for the public good.
